# Gold Nanoparticles Permit In Situ Absorbed Dose Evaluation in Boron Neutron Capture Therapy for Malignant Tumors

**DOI:** 10.3390/pharmaceutics13091490

**Published:** 2021-09-16

**Authors:** Alexander Zaboronok, Sergey Taskaev, Olga Volkova, Ludmila Mechetina, Anna Kasatova, Tatiana Sycheva, Kei Nakai, Dmitrii Kasatov, Aleksandr Makarov, Iaroslav Kolesnikov, Ivan Shchudlo, Timofey Bykov, Evgeniia Sokolova, Alexey Koshkarev, Vladimir Kanygin, Aleksandr Kichigin, Bryan J. Mathis, Eiichi Ishikawa, Akira Matsumura

**Affiliations:** 1 Department of Neurosurgery, Faculty of Medicine, University of Tsukuba, 1-1-1 Tennodai, Tsukuba 305-8575, Ibaraki, Japan; e-ishikawa@md.tsukuba.ac.jp (E.I.); matsumura.akira.ft@alumni.tsukuba.ac.jp (A.M.); 2 Department of Physics, Novosibirsk State University, 1 Pirogov Str., 630090 Novosibirsk, Russia; taskaev@inp.nsk.su (S.T.); buiya@bk.ru (E.S.); kent_brockman4@mail.ru (A.K.); kanigin@mail.ru (V.K.); 3 Budker Institute of Nuclear Physics, Siberian Branch of Russian Academy of Sciences, 11 Lavrentieva, 630090 Novosibirsk, Russia; yarullinaai@yahoo.com (A.K.); sychevatatyanav@gmail.com (T.S.); kasatovd@gmail.com (D.K.); alexxmak314@gmail.com (A.M.); ya.a.kolesnikov@inp.nsk.su (I.K.); cshudlo.i.m@gmail.com (I.S.); timaisabrony@gmail.com (T.B.); sam@211.ru (A.K.); 4 Laboratory of Immunogenetics, Institute of Molecular and Cell Biology, 8/2 Lavrentieva, 630090 Novosibirsk, Russia; volkova@mcb.nsc.ru (O.V.); lucie@mcb.nsc.ru (L.M.); 5 Department of Radiation Oncology, Faculty of Medicine, University of Tsukuba, 1-1-1 Tennodai, Tsukuba 305-8575, Ibaraki, Japan; knakai@pmrc.tsukuba.ac.jp; 6 International Medical Center, University of Tsukuba Hospital, 2-1-1 Amakubo, Tsukuba 305-8576, Ibaraki, Japan; bmathis@md.tsukuba.ac.jp

**Keywords:** boron neutron capture therapy, gold nanoparticles, dosimetry, absorbed dose, accelerator-based neutron source

## Abstract

Boron neutron capture therapy (BNCT) is an anticancer modality realized through ^10^B accumulation in tumor cells, neutron irradiation of the tumor, and decay of boron atoms with the release of alpha-particles and lithium nuclei that damage tumor cell DNA. As high-LET particle release takes place inside tumor cells absorbed dose calculations are difficult, since no essential extracellular energy is emitted. We placed gold nanoparticles inside tumor cells saturated with boron to more accurately measure the absorbed dose. T98G cells accumulated ~50 nm gold nanoparticles (AuNPs, 50 µg gold/mL) and boron-phenylalanine (BPA, 10, 20, 40 µg boron-10/mL), and were irradiated with a neutron flux of 3 × 10^8^ cm^−2^s^−1^. Gamma-rays (411 keV) emitted by AuNPs in the cells were measured by a spectrometer and the absorbed dose was calculated using the formula *D* = (*k* × *N* × *n*)/*m*, where *D* was the absorbed dose (GyE), *k*—depth-related irradiation coefficient, *N*—number of activated gold atoms, *n*—boron concentration (ppm), and *m*—the mass of gold (g). Cell survival curves were fit to the linear-quadratic (LQ) model. We found no influence from the presence of the AuNPs on BNCT efficiency. Our approach will lead to further development of combined boron and high-Z element-containing compounds, and to further adaptation of isotope scanning for BNCT dosimetry.

## 1. Introduction

Boron neutron capture therapy (BNCT) is a binary technology relying on selective boron-10 isotope (^10^B) accumulation in tumor cells (≥20 µg/g of tumor tissue) to release tumoricidal high linear energy transfer (high-LET) alpha particles and lithium (^7^Li) ions intracellularly upon irradiation with epithermal neutrons (Figure S1) [1,2,3]. This method, developed to treat invasive incurable cancers such as glioblastoma, malignant melanoma, colon, and head and neck cancers, has been proven effective in clinical trials at nuclear reactors [4,5,6,7,8,9].

Development of BNCT proceeds at a fast pace, with accelerator-based neutron sources replacing nuclear reactors, establishment of accelerator-based clinical BNCT centers in Japan and Finland, the approval of Steboronine^®^ (the first commercial boron drug), and the coverage of head and neck cancer BNCT by the Japanese national health insurance system [10,11,12,13,14,15].

Despite recent successes, several unsolved application issues remain. The unique feature of intracellular nuclear decay creates a problem when seeking to precisely measure the boron-related absorbed dose responsible for the main therapeutic effect. The short alpha-particle pathway during BNCT (~10µm) that remains within the tumor cell diameter and spares normal cells from radiation also limits dosimetry options, as there is no essential extracellular energy release and calculating the boron-related absorbed dose is difficult. For that reason, golden foils are typically used for dosimetry [16,17] and placed in proximity to tissues or samples, where they activate after irradiation and provide indirect absorbed-dose measurement by detecting 411 keV gamma-rays released from the ^198^Au isotope with a half-life of 2.69517(21) days [18]. Though widely applied, such a dosimetry method cannot provide data on actual neutron capture inside tumor cells.

**Figure 1:**
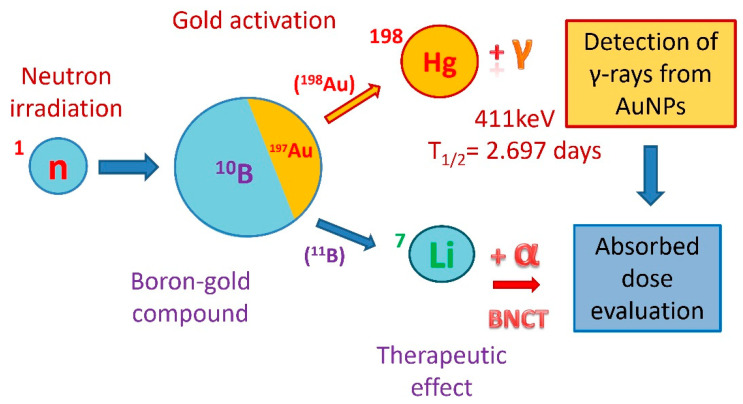
Dose evaluation concept. When a compound containing boron and gold is accumulated in tumor cells and irradiated with neutrons, along with the neutron capture reaction by boron and the therapeutic effect, gold is activated and emits 411 keV gamma-rays that can be measured and used for absorbed dose calculation.

We propose coupling gold nanoparticles (AuNPs) and boron inside tumor cells to measure the absorbed dose directly in tissues or samples based on gold activation (along with therapeutic boron neutron capture) via release of high-LET alpha-particles (Figure 1). AuNPs will become radioactive, emitting gamma-rays, and, based on this measurement plus the amount of boron and gold in samples or tissues, the boron-related absorbed dose can be more accurately estimated.

To verify the applicability of such dosimetry and initiate the development of related complex boron-gold compounds, we tested a combination of boronophenylalanine (BPA) and gold nanoparticles in human T98 glioma cells that were irradiated at an accelerator-based neutron source at the Budker Institute of Nuclear Physics in Novosibirsk Science City, Russian Federation, a prototype of a clinical accelerator by TAE Life Sciences, Inc. (Foothill Ranch, CA, USA). To the best of our knowledge, we are the first to propose such in-sample boron dose evaluation at an accelerator-based neutron source.

## 2. Materials and Methods

### 2.1. Human Glioma Cell Line

T98G cells were purchased from the Institute of Cytology of the Russian Academy of Sciences (St.-Petersburg, Russia), cultured in Iscove’s Modified Dulbecco’s Medium (IMDM) (SIGMA 17633 with L-glutamine and 25 mH HEPES, without sodium bicarbonate), supplemented with 10% fetal bovine serum (Thermo Scientific HyClone SV30160.03, HyClone UK Ltd., Cramlington, UK), and maintained at 37 °C in 5% CO_2_.

### 2.2. Fructose-BPA Solution

Analytical-grade p-boronophenylalanine (BPA) containing ≥99.6% ^10^B was purchased from Katchem Co., Ltd. (Prague, Czech Republic). The BPA-fructose solution was prepared as follows: 500 mg of BPA and 1100 mg of fructose were mixed in 15 mL of H_2_O (Milli-Q water) and 2.7 mL of 1 M NaOH, then the solution was neutralized with HCl to pH = 7.2 [19]. The resulting BPA-fructose solution (1100 µg ^10^B/mL) was diluted and added to the cell-containing medium at different concentrations.

### 2.3. Gold Nanoparticles (AuNPs)

A colloidal solution of ~50 nm glycylglycine-stabilized gold nanoparticles containing ~500 µg gold/mL was purchased from Winered Chemical, Inc. (Tokyo, Japan) and kept at 4 °C. Particle shape, size, and intracellular localization were confirmed by a JEM-1400 transmission electron microscope (TEM, JEOL, Tokyo, Japan). Gold concentration in the colloidal solution was verified by inductively coupled plasma atomic emission spectrometry (ICP-AES, ICPE-9820 simultaneous ICP atomic emission spectrometer, Shimadzu, Inc., Tokyo, Japan).

### 2.4. Cytotoxicity Assay

BPA and AuNPs cytotoxicity was evaluated by MTS assay as previously described [20]. Briefly, 100 µL of medium containing 4 × 10^4^ of T98G cells was placed in each well of 96-well plates and incubated for 24 h. The medium was exchanged with medium containing BPA (0–320 µg ^10^B/mL) or AuNPs (0–210 µg gold/mL) before were further incubation for 24 h. Then, the medium was removed and the cells were washed with PBS before a 100 µL aliquot of 2 mL of 3-(4,5-dimethylthiazol-2-yl)-5-(3-carboxymethoxyphenyl)-2-(4-sulfophenyl)-2H-tetrazolium (MTS) solution with PMS (Cell Titer 96^®^ AQueous One Solution, Promega Corporation, Madison, WI, USA) mixed into 10 mL of MEM was added to each well. The plates were further incubated for 2 h. BPA-treated cell plates were placed directly into a Bio-Rad Model 2550 EIA plate reader (Bio-Rad Inc., Hercules, CA, USA). To avoid light absorption by nanoparticles inside tumor cells, cells treated with AuNPs were transferred to a fresh well in a clean plate before measurement. For all samples, light absorption at 490 nm was measured and results are presented as ratios compared to controls without BPA or AuNPs.

### 2.5. Boron and Gold Accumulation in Tumor Cells

Boron and gold concentration in T98G cells was evaluated by ICP-AES according to previously adopted protocols [19,21]. After 24-h incubation with AuNPs (50 µg gold/mL) or BPA (10, 20, 40 µg boron/mL), the cells were washed with PBS, trypsinized, and counted. The cells were centrifuged and sedimented in polypropylene tubes, the medium was aspirated, and 1 mL of 70% HNO_3_ was added to each tube before heating to 115 °C for 2 h. In the case of AuNPs, hydrochloric acid (37% HCl) was added to form aqua regia and dissolve the gold. These solutions were diluted with Milli-Q water, filtered through 22 µm filters, and analyzed.

### 2.6. Irradiation Experiments

Neutron irradiation was carried out at the accelerator-based neutron source with a lithium neutron-producing target at the Budker Institute of Nuclear Physics (Novosibirsk, Russian Federation). This device is a prototype of a clinical BNCT facility manufactured by TAE Life Sciences, Inc. (Foothill Ranch, CA, USA) [10,13,22,23]. After 24-h-incubation with BPA containing ^10^B (0, 10, 20, 40 µg/mL) and AuNPs (50 µg gold/mL) or BPA only, the medium was removed separately for each sample and stored. To avoid BPA washout from the cells and keep similar conditions for AuNPs, the cells were suspended in 1 mL of the stored medium (with BPA or BPA and AuNPs) in which they were incubated after trypsinization. The vials were further placed in a plexiglass phantom (20 × 22 cm) at a depth of 3 cm under the neutron-producing target (Figure S2) [24,25]. The most suitable depth in the phantom was estimated based on boron and gold neutron capture cross-sections to maximize neutron capture for both elements (Figures S3–S6). The cells incubated with AuNPs (50 µg gold/mL), but without boron, and the cells incubated in medium without any of the compounds were used as controls. During irradiation, the accelerator operated with a proton current integral of 2~3 mAh and an energy of 2.0 MeV to achieve the equivalent of 6 mAh. The estimated neutron flux was 3 × 10^8^ cm^−2^s^−1^ which resulted in a total fluence of 2.16 × 10^12^ neutrons/cm^2^. At least five independent irradiation experiments were done. Neutron generation consistency was verified by a GS20 neutron detector with a lithium-containing scintillator (Saint-Gobain Crystals, Hiram, OH, USA).

### 2.7. Dosimetry and Boron Dose Calculation

**Figure 2:**
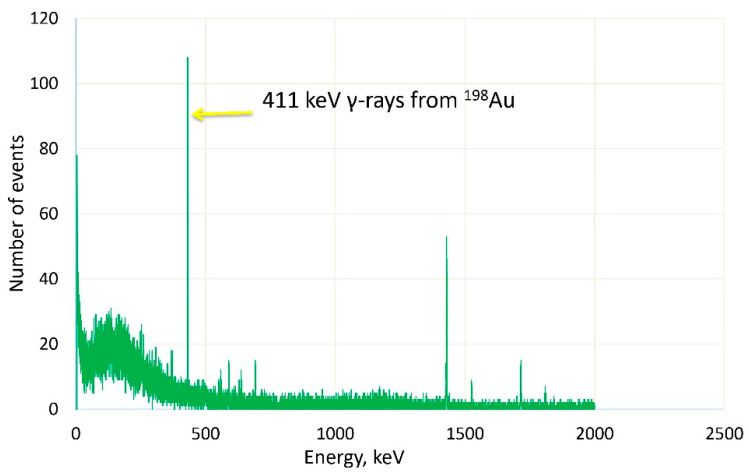
Gold activation measurement. The number of decays in time was measured by a Ge-based gamma-spectrometer. The peak at 411 keV represents the emission from the activated gold nanoparticles.

After irradiation, AuNPs-containing samples were placed under a germanium-based gamma spectrometer (HPGe, Rosatom NPO [Scientific Production Association] Centrotech, Novouralsk, Russia) [26], and 411 keV gamma-rays originating from the activated gold were separated from the background noise and measured for 100 s (Figure 2).

In creating the formula for calculating the boron-related absorbed dose, we relied on the following assumption: in theory, the speed of the ^10^B(n,α)^7^Li reaction, which is the main contributor to the BNCT dose, can be determined by registering the 478 keV gamma rays emitted by the lithium nuclei. However, the need to register gamma rays in the presence of neutrons makes this task extremely difficult in practice, while the use of activation methods makes it possible to separate the activity measurement process from the irradiation process. The ^197^Au(n,γ)^198^Au reaction is an excellent candidate for this role because it has a neutron absorption cross-section in the epithermal region similar to ^10^B (except for the resonance; Supplementary Figures S3 and S4), convenient decay time, and gamma-ray energy (411 keV) close to that of the gamma rays emitted by the lithium nucleus during boron neutron capture (478 keV).

In general, the number of reactions is proportional to the dose (*D_Au_*, *D*_10*B*_) and, in turn, depends on (1) the number of neutrons (*N*(*E*)), (2) the number of source nuclei for the reaction (*M_Au_*, *M*_10*B*_), (3) and the cross section of these reactions (*G_Au_*(*E*), *G*_10*B*_(*E*)), where E is neutron energy (E will be henceforth omitted for clarity).

After irradiation, the number of reactions that occurred in gold can be measured with a gamma spectrometer using the gold line (411 keV), meaning *D_Au_ ~ N × G_Au_ × M_Au_*, resulting in *N ~ D_Au_*/*G_Au_ × M_Au_* and then substituting in a similar formula for boron: *D*_10*B*_ *~ G*_10*B*_ *× M*_10*B*_ *× N*, *D*_10*B*_ *~ D_Au_ × G*_10*B*_ × *M*_10*B*_*/G_Au_ × M_Au_*.

A clear equality can be obtained by introducing a calculated numerical coefficient *k*, which will depend on the neutron spectrum and nuclear cross sections; given that the spectrum depends on the depth of the moderator layer, it is convenient to take the depth of the moderator layer as the parameter for the coefficient *k*.

More simply, both boron and gold are capable of capturing neutrons and, in the case of boron, this leads to the formation of the dose (*D*) while, in the case of gold, it leads to the activation (*A*). If probabilities of their neutron capture (the dependence of the cross section on the neutron energy) were similar, then *D* would equal *k* × *A* (*D = k × A*). In reality, the cross sections of these processes are different and, for the BNCT spectrum of neutrons, this leads to a small dependence of *k* on the depth *h*, which we considered in creating the formula. Thus, the dose is related to the activation by a simple relationship *D* = *k*(*h*) *× A.*

Thus, based on the assumptions, we developed the following formula to calculate the boron-related absorbed dose:

$$D=\frac{k\times Y\times nB}{nAu},$$

where *D* is the absorbed dose (*GyE*), *k*—depth-related irradiation coefficient, *Y*—sample activation (*Bq*), *nB* and *nAu*—boron and gold concentrations (*ppm*), respectively.

For convenience, we can modify the formula and take the mass, not the concentration of gold or boron, and substitute the number of formed activated gold nuclei for the number of decays of activated gold per second.

Therefore, to facilitate calculations based on equipment readings, we modified the formula as follows:

$$D=\frac{k\times N\times n}{m},$$

where *D* is the absorbed dose (*GyE*), *k*—depth-related irradiation coefficient, *N*—number of activated gold atoms, *n*—boron concentration (*ppm*), and *m*—the mass of gold (*g*).

*N* (the number of activated gold atoms) was calculated from the number of decays of activated gold atoms per second detected by the germanium gamma spectrometer (HPGe), taking into account its gold line sensitivity (411 KeV) and the half-life of activated gold (^198^Au half-life) that equals 232,675.2 s (Table 1).

The depth-related irradiation coefficient *k* and fast neutron elastic scattering-related dose were calculated using Monte Carlo simulation using the NMC code with 3D neutron transfer modeling according to the ENDF-VII cross-section database [27]. The gamma component was measured using a DVGN-01 dosimeter (Rosatom NPO [Scientific Production Association] Centrotech, Novouralsk, Russian Federation) [28].

### 2.8. Colony-Forming Assays (CF-Assays)

After irradiation, the vials were transferred to the cell laboratory and cells were washed, placed in new medium, counted, diluted, and re-seeded into 6 cm plastic dishes in the amounts of 200–4000 cells per dish, depending on the estimated irradiation dose. Two weeks after seeding, the dishes were washed with PBS, the cells were fixed with glutaraldehyde, stained with crystal violet, scanned, and colonies of ≥50 cells were counted [20]. Cell survival curves were fit to the linear-quadratic model (LQ-model) using the SOLVER add-on in Microsoft Excel (Microsoft, Inc., Redmond, WA, USA).

### 2.9. Radiobiological Parameters Calculation

Since the dose was a calculated parameter determined during the experiments and considered to be directly proportional to boron concentration in the medium, we relied on the boron concentration as it was initially set in the equations for calculating radiobiological parameters instead of the absorbed dose [25]. Thus, the linear parameter *α (alpha)* and the quadratic parameter *β (beta)* were obtained from a cell survival curve fit to the LQ-model using the equation $SF=e^{-\left(\alpha C+\beta C^{2}\right)}$ for each irradiation experiment and for all experimental data combined. With *α* and *β* parameters, boron concentration in the samples needed to control 90% of cell growth, *C*_10_, was calculated for each irradiated cell group by solving the following quadratic equation:

$$\alpha C+\beta C^{2}+ln\left(SF\right)=0,$$

where *C* represented the boron concentration (ppm) and equaled:

$$C=\frac{-\alpha \pm \sqrt{\alpha^{2}-4\beta ln\left(SF\right)}}{2\beta },$$

with positive *C*-values used.

To verify the difference in cell survival between the groups of cells irradiated with and without AuNPs, the area under the fitted curve (AUC) was calculated for each group as a definite integral of the linear-quadratic function:

$$AUC=\int_{0}^{40}\exp\left(-\alpha C-\beta C^{2}\right)dC.$$

### 2.10. Statistical Analysis

At least five independent experiments were done to analyze cell survival and at least three experiments were done to analyze other parameters. The data represent means ± standard deviations (SDs) in the case of three experiments and means ± standard errors (SEs) in the case of more than three independent experiments. Statistical significance was verified by one-way analysis of variances (ANOVA) and *p*-values ≤ 0.05 were considered indicative of statistical significance.

## 3. Results

### 3.1. AuNPs Visualization and Boron and Gold Accumulation in T98G Cells

**Figure 3:**
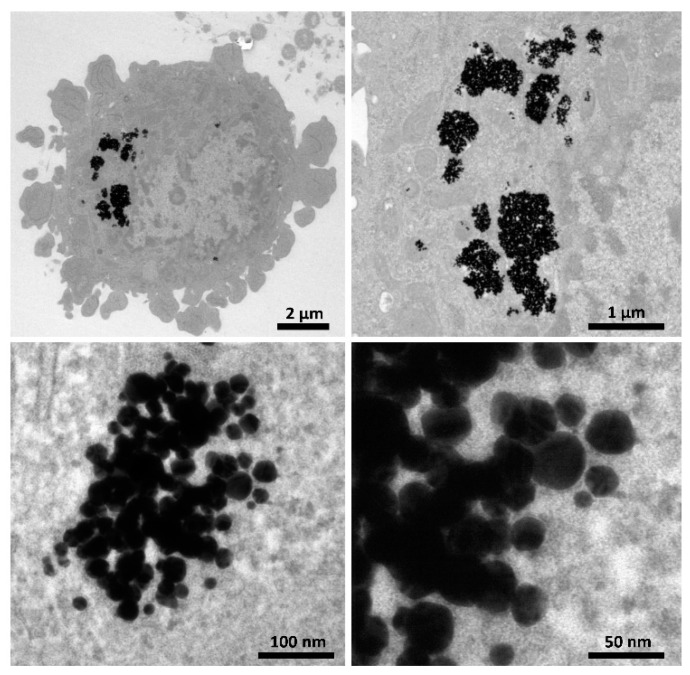
AuNPs accumulated in T98G tumor cells visualized by transmission electron microscopy (TEM). TEM images of WRGH2 AuNPs (~50 nm) in the cytoplasm of T98G cells (×2000–120,000).

On TEM images, AuNPs of variable diameter were observed in the cytoplasm of T98G cells (Figure 3). Boron and gold concentrations in the cells and medium are shown in Table 1.

### 3.2. BPA and AuNPs Cytotoxicity

**Figure 4:**
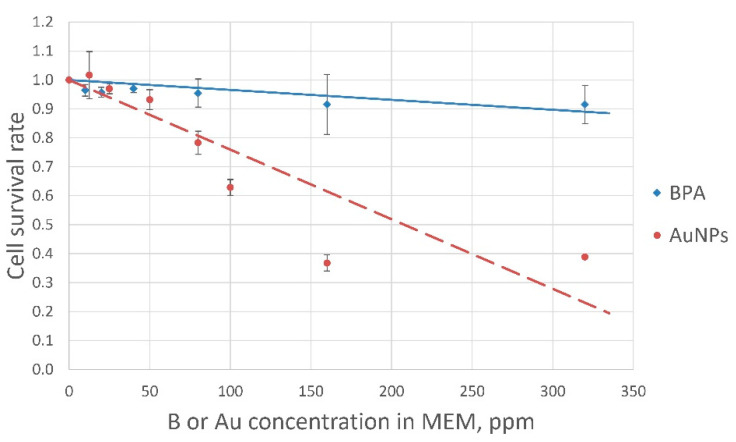
AuNPs and BPA cytotoxicity. Both compounds showed a dose-dependent proliferation response.

Both compounds showed dose-dependent cytotoxicity (Figure 4). BPA was relatively non-toxic, with a cellular proliferation rate of over 90% within the whole range of boron concentrations (0 to 320 µg/mL). In the case of AuNPs, since cell proliferation dropped at a concentration over 50 µg/mL; a 50 µg/mL maximum concentration was used in subsequent experiments.

### 3.3. Boron Dose Estimation

Activation of the AuNPs-containing samples resulted in the generation of radioactive ^198^Au isotopes that provided data for neutron dose evaluation for each boron concentration. Estimated boron-related absorbed doses are shown in Table 1. Detailed calculations can be seen in Table S1.

### 3.4. Glioma Cell Survival after Neutron Irradiation with and without AuNPs

**Figure 5:**
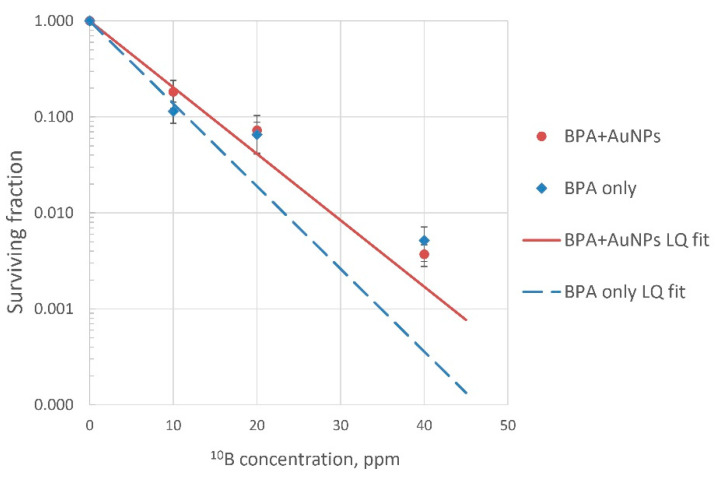
T98G cell survival after BNCT. T98G cells were treated with BPA (B10: 0–40 ppm) and irradiated with neutrons (fluence: 2.16 × 10^12^ n/cm^2^) with or without AuNPs (Au: 50 ppm).

CF assays confirmed BNCT efficacy; T98G cell survival curves are presented in Figure 5. BNCT decreased tumor cell survival exponentially depending on boron concentration with the most significant BNCT effect at boron concentrations of 40 µg/mL. The obtained cell survival data were in line with previously published reports on in vitro BNCT experiments using other cell lines [25,29,30].

Radiobiological parameters are shown in Table 2. The presence of AuNPs in the samples did not significantly influence cell survival.

## 4. Discussion

Radiation dose distribution analysis in relation to BNCT treatment planning traditionally uses complex equations to summarize the effects of all dose components [31,32,33]. However, depending on the irradiation source and neutron beam properties, the actual absorbed dose in BNCT is related to several separate components that include: effects occurring within irradiated tumor cells; atomic interreactions within tumor and normal tissues (high-LET and low-LET radiation from boron neutron capture reactions releasing α and ^7^Li particles, hydrogen and fast and epithermal neutron-related recoil protons, nitrogen capture-related protons and hydrogen capture-related gamma rays); incident radiation effects of thermal, epithermal and fast neutrons; neutron source- and capture-related gamma rays; and inelastic neutron scattering in the beam-shaping assembly [34]. Computations of the impact of these phenomena on different biological tissues using different radiobiological effectiveness coefficients is possible only through complex computer simulation programs [35,36,37] and, with the development of new accelerator-based neutron sources, irradiation dose evaluation in a clinical environment requires more direct measurement to ensure dosage precision.

Here, we propose a convenient and direct way to estimate the boron-related absorbed dose responsible for the main therapeutic effect through extracellular radioemission. In contrast, the traditional system relies on calculations only; the actual dose from momentary nuclear reactions within biological tissues may only be estimated since no radioactivity clears the cellular barrier for actual measurement. We admit that, in our calculations, we rely on the *k*-coefficient, a depth-related parameter, as assessed by Monte Carlo simulation. Therefore, while it is difficult to completely separate our method from computer simulations, our approach gives insight into intracellular reactions that may assist in predicting both therapeutic effect and the necessity of further irradiation.

We focused on estimating the boron dose while classifying the impact of other significant irradiation as the sum of the three doses (GyE) (from nitrogen activation, fast neutrons, and gamma radiation) and equal to 20% of the boron dose at 40 ppm boron concentration in the sample, as previously reported [23]. As the precise calculation of dose components other than the boron-related one was beyond the purpose of this study, we used cells irradiated without boron as a control so that the survival curves reveal the isolated effect of boron (Figure 5).

Table 1 summarizes the main parameters used in the calculations to determine the boron-related absorbed dose and individual values for each experiment, along with the average values. The samples were divided into three groups depending on initial boron concentration; however, the final concentration in the samples during experiments differed not only depending on the initial boron concentration but also on the number of cells in each sample and their ability to accumulate boron. The formulas for boron-related absorbed dose calculation were initially described in the Materials and Methods and they are additionally given in the notes to the table (Table 1). These experiments showed that the total amount of ^10^B in the samples, as well as gold, depended on cellular accumulation. Boron concentrations in our experiments were relatively low, though in line with another report on BPA accumulation in T98G cells [38]. In contrast, AuNPs were taken up by cells in much larger amounts, an effect possibly related to the complexities of intracellular transport as seen in BPA, a small molecule that relies on amino acid transporters including ATB(0,+) and L-amino acid transporters 1 and 2 (LAT-1 and LAT-2, respectively) [39,40], as well as in nanoparticles reliant upon endocytosis [41,42]

As BPA tends to wash out over time in exchange for other amino acids, intracellular boron concentrations were maintained by steady-state BPA concentration in the medium during the experiments. Similar conditions were used for AuNPs, although nanoparticle release from tumor cells was not expected and this may have led to high accumulation within cells. Thus, the total amount of both boron and gold in the samples was determined as the sum of the intracellular concentration and the residual concentration in the medium after incubation. Readings from the germanium gamma-spectrometer, corrected based on its sensitivity in the gold line (411 keV), determined the number of decays of activated gold atoms per second and were used to calculate the number of activated gold atoms in each of the experiments. Based on the calculations, the absorbed doses were directly proportional to the amounts of boron in the samples, as initially assumed, and Figure 5 displays this BNCT effect.

In Table 2, the cell survival data with regard to boron concentration and the calculated radiobiological parameters are shown. As expected, the fraction of cell survival was inversely proportional to the concentration of boron in the cells which, in turn, determined the amount of alpha irradiation of the cells and corresponded to an increase in the boron-dependent absorbed dose. In four out of five experiments and in the overall fit, the *β* parameter equaled 0 (zero), showing a linear decrease in cell survival due to high-LET radiation effects. In one experiment in each set (with and without AuNPs), *β* differed from zero, likely due to other factors; however, these *β* values were several orders of magnitude lower (trending towards zero) than those typical for cellular exposure to low-LET radiation. The effect of gold on cellular survival was ruled out by preliminary experiments to set a maximum tolerance, but future experiments may require lower gold concentrations to remove any trace of interference from boron-related dose absorption studies. As intracellular boron concentration was relatively low, the lethality of the BNCT performed in this study could be due to higher medium concentrations modeling boron accumulation in extracellular compartments, rendering the external media as a source of BNCT-generated damage to the membranes of tumor cells [20]. However, achieving a “perfect” BNCT effect on tumor cells was beyond the main purpose of this study, which was to use gold nanoparticles to increase the precision of radiation dose measurement.

In our study we used AuNPs with an average size of ~50 nm, as such gold particles were found to bioaccumulate at higher concentrations than AuNPs of other sizes [42]. AuNPs cytotoxicity is generally considered to be low, but the higher AuNPs cytotoxicity in our case could be related to low culture medium concentrations that influenced cell nutrition and decreased proliferation when larger amounts of colloidal gold were added. Another issue might be related to nanoparticle uptake and concentration within the cellular cytoplasm (Figure 3) that could hinder cell division. Therefore, in irradiation experiments, we used a lower AuNPs concentration (50 µg/mL) that resulted in a cellular proliferation rate of over 90% after incubation, and then eliminated that influence by maintaining concentrations in the experimental gold-containing samples and corresponding controls.

As a high-Z element, gold can absorb radiation and, in our study, its ability to capture neutrons along with boron could have caused a shielding effect in tumor cells. On the other hand, the emission of secondary photons or electrons from AuNPs after their activation by neutrons or other types of radiation during BNCT could add a portion of non-LET radiation and influence cell survival [43]. Though the overall exponential decrease in surviving fractions between the groups of cells irradiated with and without AuNPs looked different with the elevated fitted curve for the AuNPs-group (Figure 5), further analysis revealed no statistical differences from cells irradiated without AuNPs. Therefore, we concluded that even excessive gold in tumor cells had no critical impact on cellular survival and thus did not influence the results of our experiments.

Thus, we found that our boron-gold combination was useful to estimate the boron-dependent absorbed dose while the gold component had no real influence on the actual BNCT process or culture cytotoxicity. Prospective complex boron-gold compounds can thus use sufficient gold to avoid any possible shielding or side effects due to undesirable additional radiation emission while remaining suitable for boron dose evaluation. Additionally, because the half-life of the activated gold is on the order of days, such dosimetry could be done at a relaxed pace after BNCT is completed.

This study is a proof of concept for the use of both boron and gold for theranostic purposes, and it is clear that transferring these experiments to laboratory animals requires a novel boron-gold compound that will possess active tumor-targeting properties. The enhanced permeability and retention (EPR) effect [44], described as a way of passive tumor targeting due to fenestrated capillaries in tumor tissue, might work in some cases but cannot provide compound delivery in the vast variety of malignances. Thus, specific, active tumor targeting at a molecular level is required to successfully deliver therapeutic agents to tumor cells. Therefore, we will face certain challenges when transferring our experiments to the in vivo level.

Since membrane trafficking of small molecules and nanoparticles differs [39,40,41,42], a more reliable way to deliver larger amounts of both diagnostic and therapeutic components might be realized through combinations of gold and boron in single nanoparticles, making it easier to track them in the body and to determine pharmacokinetics, pharmacodynamics, and tolerability as well as therapeutic and diagnostic effectiveness after neutron irradiation. As non-covalently combined substances (e.g., chelated compounds) can be detached from each other and transported separately (binding to blood proteins) while maintaining stability in aqueous solutions [21,45], covalent binding of boron and gold or binding in one crystal lattice might be necessary for such complex compounds.

One critical issue for in vivo use is the administration method since, in relation to the EPR effect, intravenously injected nanoparticles might primarily accumulate in organs with fenestrated capillaries such as the liver and spleen. Therefore, a more rational option would be an intra-arterial injection of nanoparticles through the arteries feeding the tumor, which would reduce the amount of injected drug and improve targeted delivery. This method has been used in clinical trials [46,47,48], and, in our opinion, seems to be the most rational. On the other hand, intra-arterial injection of nanoparticles carries risks of increased nanoparticle binding to blood components and can potentially lead to thrombi or stenosis. Moreover, during the therapy, circulating nanoparticles may interact with the kidneys [49,50], leading to a worst-case renal tubule blockage. Accordingly, a prerequisite for the preparation of nanoparticles will be the maximum delineation of nanoparticle sizes that are safe for administration to animals and, subsequently, to humans. Therefore, a more detailed study of this issue is necessary when developing the method of nanoparticle administration.

The most important point to develop successful tumor-targeting complex nanoparticles will be specific binding to receptor molecules on tumor cell membranes or inside tumor cells, sequestering the active components during both therapeutic irradiation and afterwards to adequately measure gamma rays from the activated gold atoms. At the moment, our research group is in the process of developing such a novel boron-gold compound with active tumor targeting properties that can be further applied in experiments in vivo.

Other, previously reported boron-gold compounds exist such as those by Mandal et al. (2011), who developed structures of gold nanoparticles coated with polyelectrolytes containing fluorescent dye (FITC), BPA, and folic acid as a boron delivery agent [51]. Wu et al. (2019) developed anti-HER2 antibody-conjugated theranostic boron-containing PEGylated AuNPs that were labeled with ^123^I and detectable by single-photon emission computed tomography (SPECT) and computed tomography (CT) [52]. However, the purpose of these studies was different from ours, which was to isolate the effects of gold and boron on cellular parameters and radiation dosage measurements that are relevant to clinical BNCT. Thus, the challenge of fusing gold and boron into BNCT-specific nanoparticles is a future technical issue beyond the scope of this preliminary study but, even with separate use of BPA and AuNPs, the low error in our method was confirmed to be useful in boron dose estimation.

In the future, activated gold might be considered as a radionuclide for isotope scanning, a method of clinical diagnosis that has been used for decades [53], and, for that purpose, the scanning sensor needs to be adjusted to the necessary energy of gamma rays emitted by activated gold. Thus, the development of the proposed BNCT dosimetry approach might also provide detection of tumor localization by building composite images of boron- and gold-saturated tumor cells, leading to further utilization of this method in isotope scanning after its appropriate modification for BNCT needs.

## 5. Conclusions

We tested a new approach for BNCT absorbed dose evaluation using gold nanoparticles placed in tumor cells and proved its effectiveness in postradiation dosimetry. Our method, based on gold activation by neutron irradiation with further measurement of gold-emitted gamma rays, has no significant deleterious effects on the radiotherapy but allows for dosimetry for hours or days after irradiation. Our results support further development of combined boron-gold compounds that may open up new perspectives in the evaluation of drug distribution, activation in tumor tissues, and adjustment of isotope scanning that might improve related diagnostic methods and further development of BNCT.

## 6. Patent

Taskaev S.; Zaboronok A. Method of measuring absorbed dose in boron neutron capture therapy. Patent for invention No. 2606337, 25 November 2015.

## Figures and Tables

**Table 1:** Boron dose calculation.

Samp.	Exp.	Initial B^10^/Au Conc. in MEM, ppm	Cell Count, N × 10^6^	B^10^ Concentration, ppm			Mass of Gold, µg			HPGe Reading Value in Gold Line	Decays per Second	Number of Activated Gold Atoms	Boron Dose per Sample, GyE	Mean Boron Dose in Samp., GyE ± SD
				Cells	1 mL MEM	Samp.	Cells	1 mL MEM	Samp.					
B10 Au+	1	10/50	3.0	0.148	9.95	10.10	140.83	3.06	143.89	12.9	274.47	92,133,408	4.80	5.45 ± 0.73
	2		1.7	0.084	9.97	10.06	79.80	23.40	103.20	10.3	219.15	73,563,884	5.32	
	3		1.4	0.069	9.98	10.05	65.72	28.09	93.81	11.0	234.04	78,563,371	6.24	
B20 Au+	1	20/50	2.15	0.148	19.95	20.10	92.91	19.03	111.94	13.3	282.98	94,990,257	12.65	9.96 ± 2.67
	2		2.1	0.145	19.95	20.10	90.75	19.75	110.50	10.3	219.15	73,563,884	9.93	
	3		1.75	0.120	19.96	20.08	75.63	24.79	100.42	6.9	146.81	49,280,660	7.31	
B40 Au+	1	40/50	2.9	0.549	39.82	40.37	140.78	3.07	143.85	12.9	274.47	92,133,408	19.18	18.56 ± 1.96
	2		2.0	0.379	39.87	40.25	97.09	17.64	114.73	8.8	187.23	62,850,697	16.36	
	3		1.1	0.208	39.93	40.14	53.40	32.20	85.60	8.1	172.34	57,851,209	20.13	

Abbreviations: Samp.—samples, Exp.—experiment, conc.—concentration, MEM—medium, HPGe—germanium-based gamma spectrometer [26], GyE—gray equivalent, SD—standard deviation. HPGe reading values in gold line were absolute numbers, HPGe sensitivity in gold line (411 KeV) = 0.047, number of decays per second = HPGe reading values/sensitivity, Au^198^ half-life = 232,675.2 s, number of activated gold atoms = number of decays per second/(1 − 2^(−1/Au198 half-life)^). Boron dose calculation formula: *D* = (*k* × *N* × *n*)/*m*, where *D*—the absorbed dose (GyE), *k*—depth-related irradiation coefficient, *N*—number of activated gold atoms, *n*—boron concentration (ppm), and *m*—the mass of gold (g). In case of three independent experiments, SDs were used.

**Table 2:** Radiobiological parameters.

Samp.	Initial B^10^/Au Conc. in MEM, ppm	Cell Surviving Fraction, SF ± SE	α, Mean ± SE (Overall Fit)	β, Mean ± SE (Overall Fit)	*p*-Values (α/β)	*C*_10_, Mean ± SE (Overall Fit)	*p*-Value (*C*^10^)	Area under Curve, Mean ± SE (Overall Fit)	*p*-Value (AUC)
B0 Au+	0/50	1.000 ± 0.000	0.1912 ± 0.0421 (0.1594)	0.0002 ± 0.0002 (0)	0.806 (α) / 0.432 (β)	15.8188 ± 2.4072 (14.4455)	0.112	6.1294 ± 1.2705 (6.2629)	0.395
B10 Au+	10/50	0.182 ± 0.058							
B20 Au+	20/50	0.072 ± 0.031							
B40 Au+	40/50	0.004 ± 0.001							
B0 Au-	0/0	1.000 ± 0.000	0.2043 ± 0.0297 (0.1982)	0.0011 ± 0.0011 (0)		11.0632 ± 1.1490 (11.6151)		4.8937 ± 0.5277 (5.0426)	
B10 Au-	10/0	0.114 ± 0.028							
B20 Au-	20/0	0.065 ± 0.023							
B40 Au-	40/0	0.005 ± 0.002							

Abbreviations: Samp.—samples, conc.—concentration, MEM—medium, SF—surviving fraction, SE—standard error, AUC—area under curve, C_10_—B^10^ concentration in the medium needed to eliminate 90% of tumor cells (achieve 10% of tumor cell survival). In case of five or more independent experiments, SEs were used.

## Data Availability

The data presented in this study are available on request from the corresponding author.

## Supplementary Materials

The following are available online at https://www.mdpi.com/article/10.3390/pharmaceutics13091490/s1, Figure S1: Principle of boron neutron capture therapy (BNCT), Figure S2: Combined compound irradiation, Figure S3: ^10^B (n,α) 7Li cross section, Figure S4: ^197^Au radioactive capture cross section, Figure S5: *k*—coefficient values versus depth of the plexiglas, Figure S6: Evaluation of the most suitable depth in plexiglass depending on both boron neutron capture reaction and gold activation, Table S1: Boron-related absorbed dose calculation.
