# Supplementary material for: Gold Nanoparticles Permit In Situ Absorbed Dose Evaluation in Boron Neutron Capture Therapy for Malignant Tumors
Source: Pharmaceutics. 2021 Sep 16;13(9):1490. doi: 10.3390/pharmaceutics13091490 (PMC8466622; doi:10.3390/pharmaceutics13091490)
Supplement: Supplementary file 1 [file pharmaceutics-13-01490-s001.zip › pharmaceutics-1355843-SM.pdf]

pharmaceutics

Supplementary Materials: Gold Nanoparticles Permit in Situ Absorbed Dose Evaluation in Boron Neutron Capture Therapy for Malignant Tumors

Alexander Zaboronok, Sergey Taskaev, Olga Volkova, Ludmila Mechetina, Anna Kasatova, Tatiana Sycheva, Kei Nakai, Dmitrii Kasatov, Aleksandr Makarov, Iaroslav Kolesnikov, Ivan Shchudlo, Timofey Bykov, Evgeniia Sokolova, Alexey Koshkarev, Vladimir Kanygin, Aleksandr Kichigin, Bryan J. Mathis, Eiichi Ishikawa and Akira Matsumura

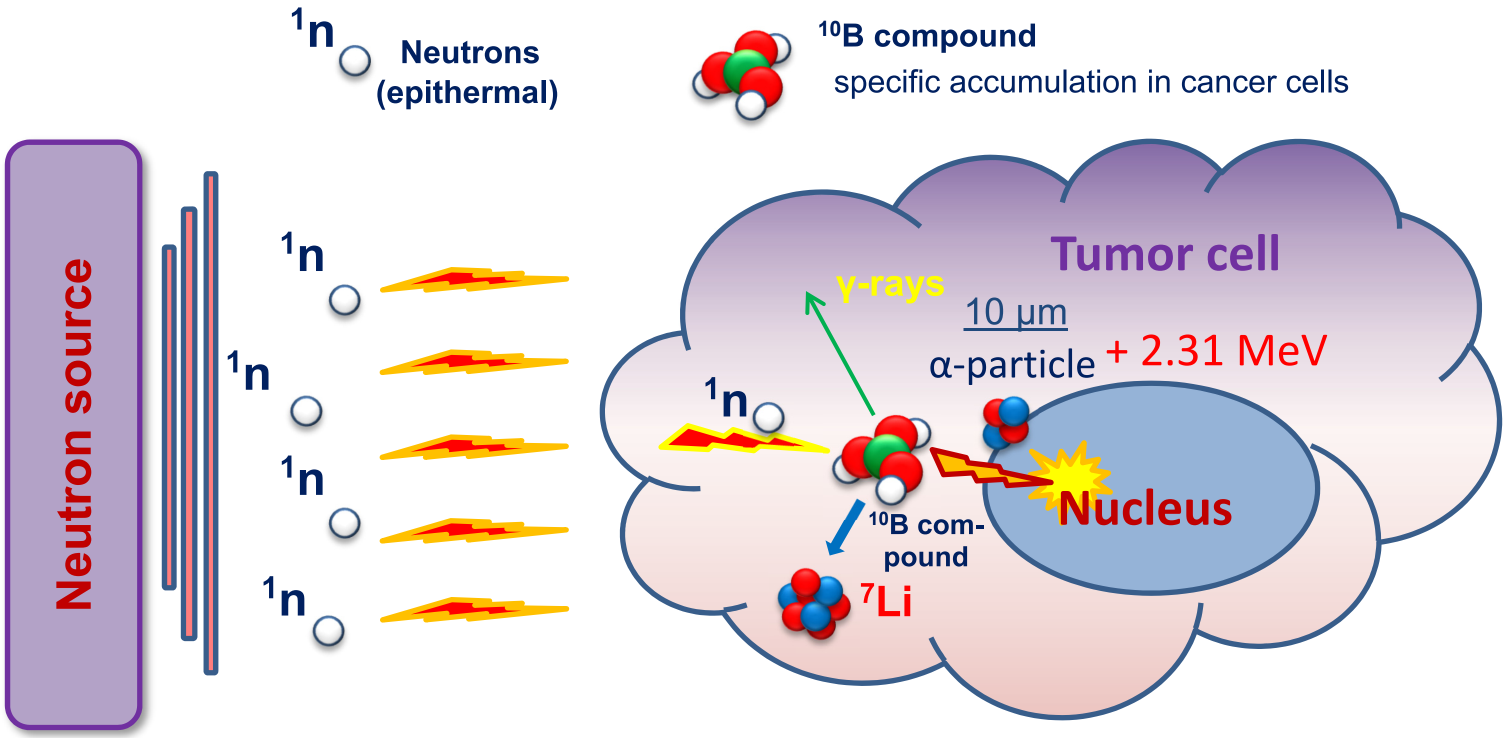

Figure S1. Principle of boron neutron capture therapy (BNCT). BNCT is a binary technology that is realized through boron-10 isotope accumulation in tumor cells and further irradiation of the tumor area with epithermal neutrons, leading to intracellular boron neutron capture reaction ($^{10}B(n,\alpha)^{7}Li$) resulting in the release of high-LET alpha particles and lithium (^{7}Li) ions that damage tumor cell DNA.

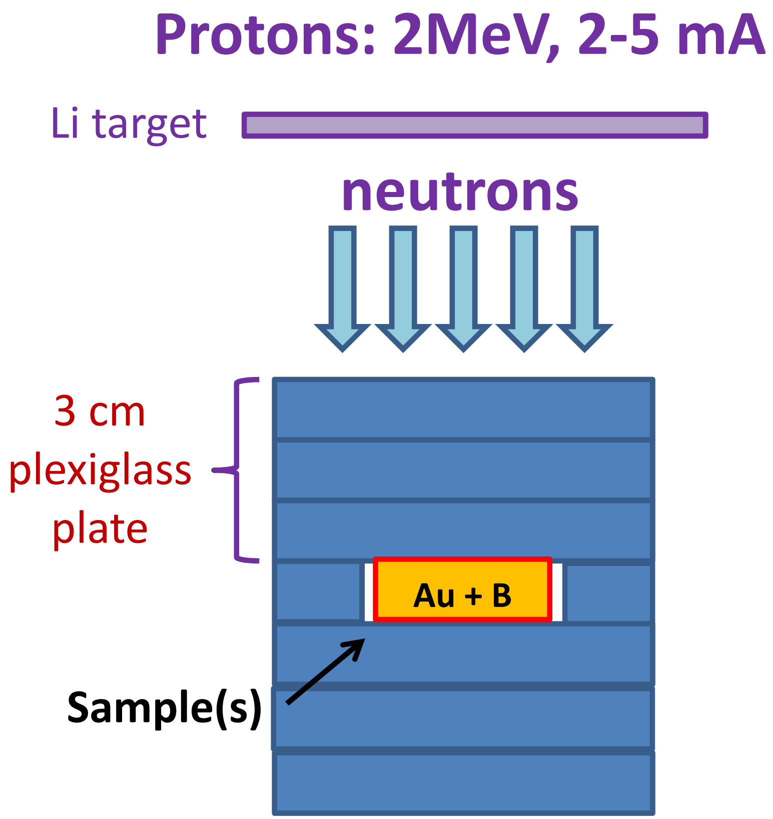

Figure S2. Combined compound irradiation. Cell-containing vials are placed in a plexiglass phantom (20 x 22 cm) at the depth of 3 cm under the neutron-producing target and irradiated with epithermal neutrons.

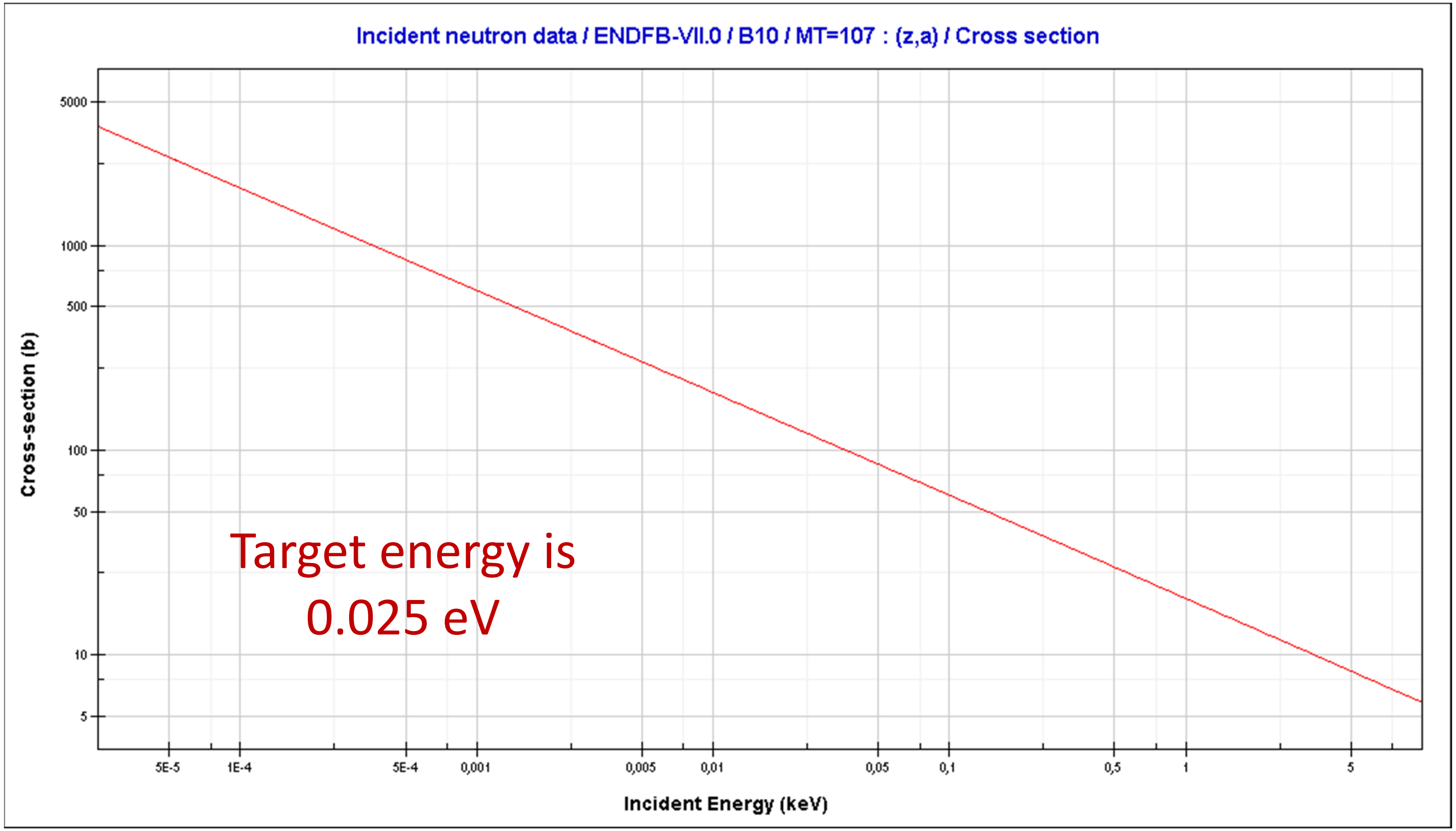

Figure S3. ^{10}B (n,α) ^{7}Li cross section.

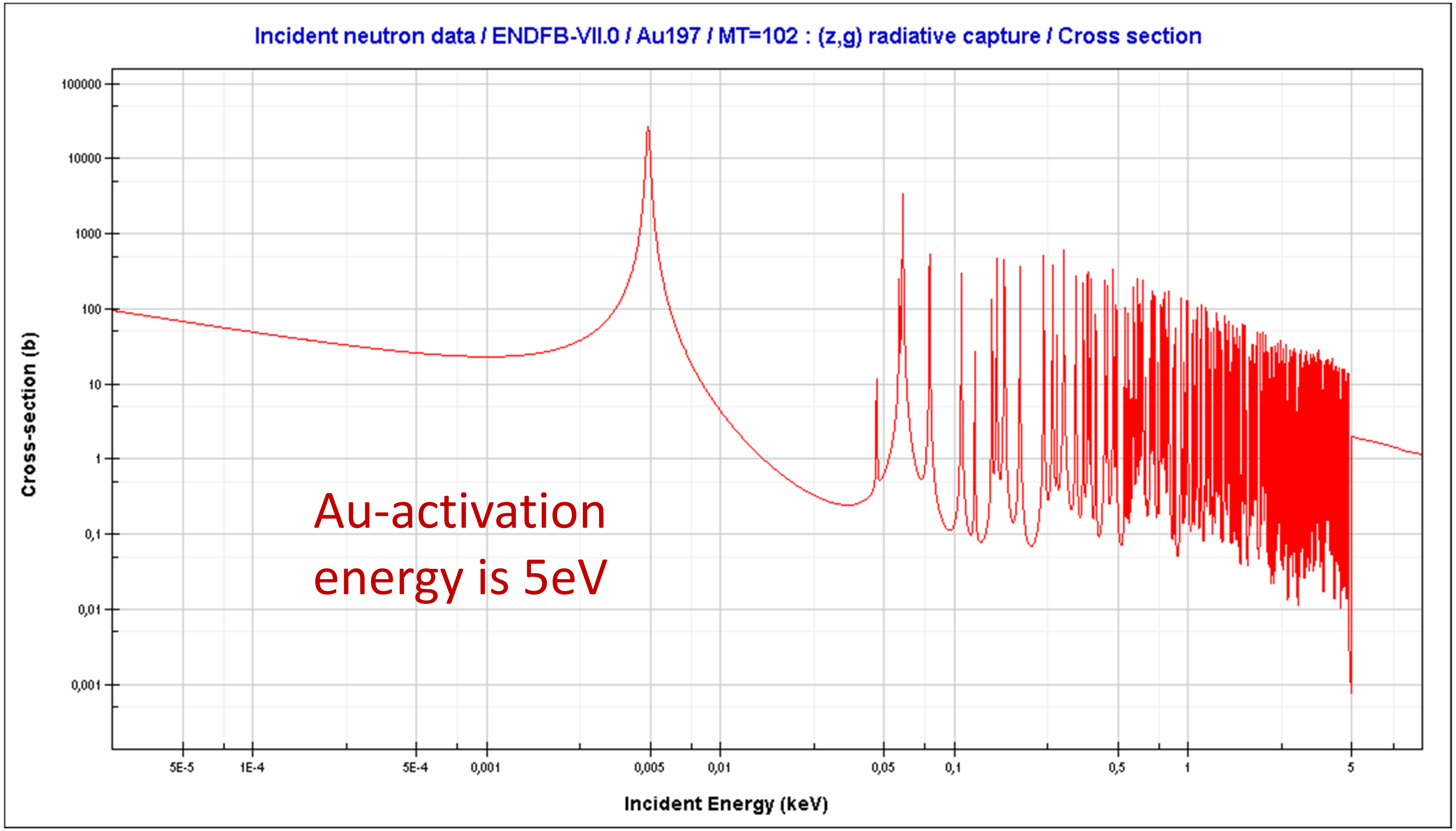

Figure S4. ^{197}Au radioactive capture cross section.

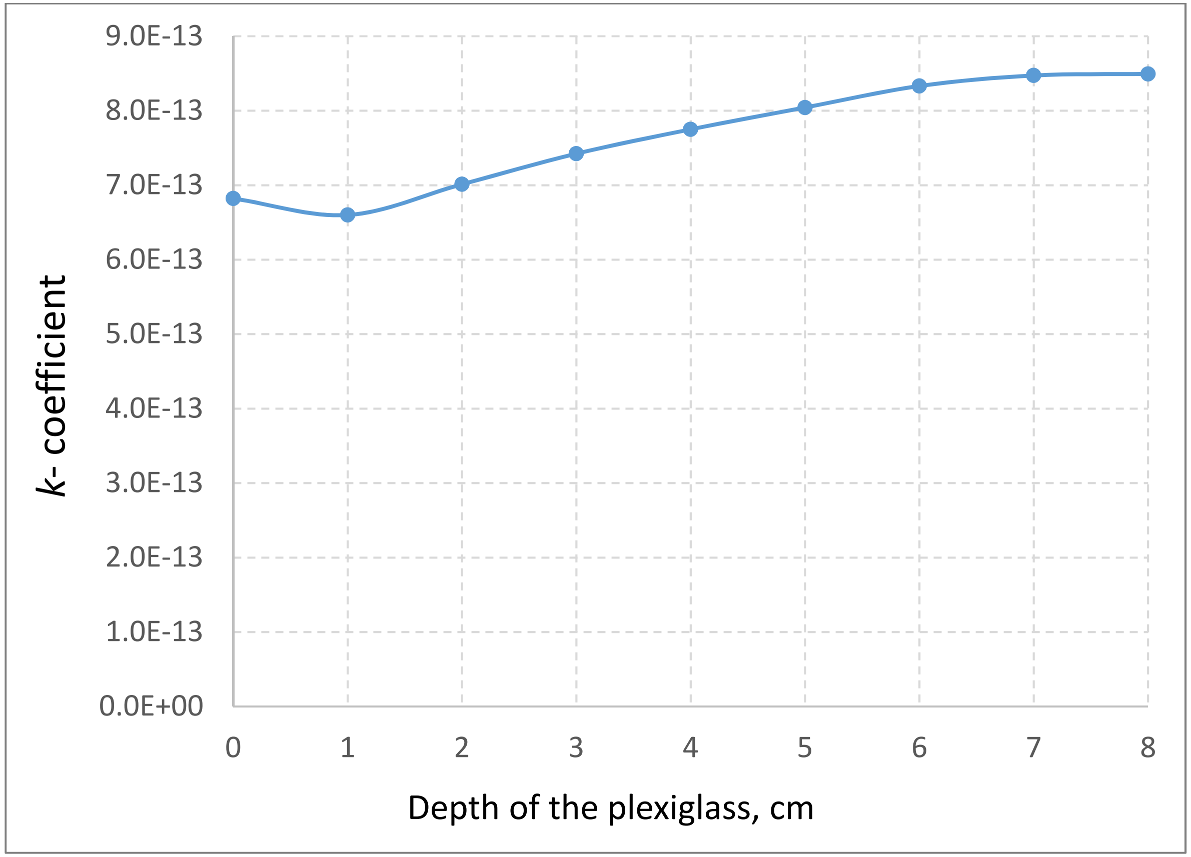

Figure S5. *k* – coefficient values versus depth of the plexiglas. The depth-related irradiation coefficient was calculated by Monte Carlo method calculated using the NMC code with 3D neutron transfer modeling according to ENDF-VII cross-section database.

Depth (cm)	Dose from boron -10 (Gy/min)	Reaction rate in 1g of Au
0,4	2,59841	9,36E+11
0,8	3,34421	9,39E+11
1,2	3,79428	8,65E+11
1,6	4,06395	7,71E+11
2	4,20305	6,75E+11
2,4	4,239	5,82E+11
2,8	4,20214	4,97E+11
3,2	4,0982	4,20E+11
3,6	3,93107	3,51E+11
4	3,72389	2,92E+11
4,4	3,49357	2,39E+11
4,8	3,25098	1,96E+11
5,2	2,98715	1,60E+11
5,6	2,72229	1,28E+11
6	2,4771	1,03E+11

Figure S6. Evaluation of the most suitable depth in plexiglass depending on both boron neutron capture reaction and gold activation.

Table S1. Detailed calculations of estimated boron-related absorbed doses.

Cells	Initial B conc., ppm	Number of cells, $\times 10^6$	Boron concentration, ppm: In 10^6 cells	In all cells	In 1mL MEM	In cells + MEM	Mass of gold: In 10^6 cells, μg	In 1mL MEM, μg	In all cells, μg	In cells+MEM, μg	Convert to g	Count rate in gold line by HPGe	Decays per second	N – number of activated gold atoms	D=(k×N×n)/m Boron dose in samples, GyE
B- Au+	0	2.75	0	0	0	0	47.57	6.39	130.82	137.21	1.37×10^4	10	212.77	71421246	0
B10 Au+	10	3	0.049	0.148	9.95	10.10	46.94	3.06	140.83	143.89	1.44×10^4	12.9	274.47	92133408	4.80
B20 Au+	20	2.15	0.069	0.148	19.95	20.10	43.22	19.03	92.91	111.94	1.12×10^4	13.3	282.98	94990257	12.65
B40 Au+	40	2.9	0.189	0.549	39.82	40.37	48.54	3.07	140.78	143.85	1.44×10^4	12.9	274.47	92133408	19.18
B- Au+	0	1.25	0	0	0	0	47.57	30.18	59.46	89.64	8.96×10^5	12.9	274.47	92133408	0
B10 Au+	10	1.7	0.049	0.084	9.97	10.06	46.94	23.40	79.80	103.20	1.03×10^4	10.3	219.15	73563884	5.32
B20 Au+	20	2.1	0.069	0.145	19.95	20.10	43.22	19.75	90.75	110.50	1.11×10^4	10.3	219.15	73563884	9.93
B40 Au+	40	2	0.189	0.379	39.87	40.25	48.54	17.64	97.09	114.73	1.15×10^4	8.8	187.23	62850697	16.36
B- Au+	0	1.6	0	0	0	0	47.57	24.63	76.11	100.74	1.01×10^4	11.9	253.19	84991283	0
B10 Au+	10	1.4	0.049	0.069	9.98	10.05	46.94	28.09	65.72	93.81	9.38×10^5	11	234.04	78563371	6.24
B20 Au+	20	1.75	0.069	0.120	19.96	20.08	43.22	24.79	75.63	100.42	1.00×10^4	6.9	146.81	49280660	7.31
B40 Au+	40	1.1	0.189	0.208	39.93	40.14	48.54	32.20	53.40	85.60	8.56×10^5	8.1	172.34	57851209	20.13

D – boron dose, GyE

N – number of activated gold atoms

n – boron concentration, ppm

m – mass of gold, g

k – coefficient, which depends on the depth (cm) to the sample in the phantom

Sensetivity HPGe in gold line (411KeV) 0.047

The time of ^{198}Au half-life, s 232675.2

k= 7.4×10^{-13}

	Cells/Exp	B- Au+	B10 Au+	B20 Au+	B40 Au+
Boron dose in samples, GyE	1	0	4.80	12.65	19.18
	2	0	5.32	9.93	16.36
	3	0	6.24	7.31	20.13
AVERAGE		**0**	**5.45**	**9.96**	**18.56**
SD		0	0.73	2.67	1.96
